# Resveratrol ameliorates myocardial fibrosis by regulating Sirt1/Smad3 deacetylation pathway in rat model with dilated cardiomyopathy

**DOI:** 10.1186/s12872-021-02401-y

**Published:** 2022-01-26

**Authors:** Qingquan Chen, Yu Zeng, Xiulin Yang, Yue Wu, Shuyu Zhang, Shirong Huang, Yameng Zhong, Min Chen

**Affiliations:** 1 Department of Laboratory Medicine, School of Medical Technology and Engineering, Fujian Medical University, 88 Jiaotong Road, Fuzhou, 350004 Fujian China; 2 Xiamen Maternal and Pediatric Hospital, Women and Children’s Hospital Affiliated To Xiamen University, Xiamen, 361003 China; 3 Department of Laboratory Medicine, Fujian Obstetrics and Gynecology Hospital, Fuzhou, 350012 China; 4 The Key Laboratory of Fujian Province Universities on Ion Channel and Signal Transduction in Cardiovascular Diseases, Fuzhou, 350122 China

**Keywords:** Resveratrol, Fibrosis, Sirt1, Smad3, Dilated cardiomyopathy

## Abstract

**Background:**

The aim of this study was to investigate the effects of Resveratrol (RSV) in rats with dilated cardiomyopathy (DCM).

**Methods:**

Porcine cardiac myosin was used to set up rat model with DCM. RSV (10 mg/kg in RSV-L group and 50 mg/kg in RSV-H group) or vehicle was administered to rats with DCM once daily from the 28th day till the 90th day after the first immunization. Cardiac function of rats was evaluated by echocardiographic analysis. The deposition of fibrous tissues in the hearts was evaluated by Masson and picrosirius red staining. The mRNA levels of collagen type I (Col I), collagen type III (Col III) and silence information regulator 1 (Sirt1) were measured by quantitative real-time polymerase chain reaction (qRT-PCR). The interaction of Sirt1 with Smad3 was revealed by coimmunoprecipitation.

**Results:**

The heart weight, heart weight/body weight ratio, left ventricular end diastolic diameter (LVEDD) and left ventricular end systolic diameter (LVESD) were significantly increased in rats with DCM, and attenuated by RSV. RSV also positively decreased fibrosis, and the expression of Col I and Col III in the myocardium. The Sirt1 mRNA was significantly decreased in myosin-immunized hearts and was positively increased by RSV. The Sirt1 combined with Smad3 directly. Acetylation of Smad3 (Ac-Smad3) was significantly increased in DCM and was markedly decreased by RSV.

**Conclusion:**

RSV effectively ameliorated myocardial fibrosis and improved cardiac function by regulating Sirt1/Smad3 deacetylation pathway in rat model with DCM.

## Introduction

Dilated cardiomyopathy (DCM) is mainly characterized by complex remodeling of one or both ventricles with an associated increase in mass, volume and the architecture of the myocardium fibres, resulting in left ventricular systolic dysfunction. It is the most common non-ischemic cardiomyopathy throughout the world, with an estimated prevalence of 1:2500–1:250 in the general population [1–3]. DCM can be caused by many risk factors, such as hypertension, inflammation, infection, valve disease, metabolic and toxic effects medications [4]. It also commonly has an underlying genetic variation which accounts for 30–48% of cases with DCM [3]. Affected individuals are at risk of heart failure, sudden cardiac death and other life-threatening risks. In DCM, myocardial fibrosis, which plays a vital role in the genesis of ventricular arrhythmias, is known as an important pathophysiological process [5]. Myocardial fibrosis can also be used to predict ventricular arrhythmias and sudden cardiac death in patients with nonischemic DCM [6].

The acetylation of Smad3 (Ac-Smad3) level was high in the rats with cardiac fibrosis [7, 8] and renal fibrosis [9], while it was low in the normal myocardium and nephridial tissue of rats. Ac-Smad3 can regulate Smad3 DNA binding activity and transcriptional activity of specific profibrotic genes [10, 11]. So, increasing Ac-Smad3 level by transforming growth factor-beta 1 (TGF-β1) promote the occurrence and development of tissue fibrosis [10, 12]. Accordingly, collagen lattice contraction were impaired in Smad3−/− fibroblasts, and collagen deposition in the infarcted heart was reduced in Smad3 null mice [13], which reflects decreasing Ac-Smad3 level in Smad3−/− fibroblasts or Smad3 null mice will prevent or attenuate the tissue fibrosis. Therefore, the Ac-Smad3 level may play an important role in the tissue fibrosis. Recently, studies found that Ac-Smad3 can be targeted by resveratrol (RSV) [14], geniposide [8], metformin [15], carnosic acid [16] and nicotinamide riboside [17] to ameliorate the tissue fibrosis. The Ac-Smad3 level was mainly adjusted by the activation of acetyltransferase or histone deacetylase. There are two solutions to reduce the level of Ac-Smad3. One is to reduce the activity of acetyltransferase, such as lysine acetyltransferase 5 can be suppressed by metformin [15] to decreased the Ac-Smad3 level, the other is to enhance the activation of histone deacetylase, such as the activation of histone deacetylase silence information regulator 1 (Sirt1) can be increased by resveratrol (RSV) [14], geniposide[8], carnosic acid [16] and nicotinamide riboside [17] to decreased the Ac-Smad3 level.

As a natural non-flavonoid polyphenol, resveratrol (RSV) found in grapes and other plants, is a natural activator for Sirt1 [18]. Li J, et al. [9] found that RSV can inhibit renal fibrosis by the activation of Sirt1, which mediated the deacetylation of Smad3 and suppressed the TGF-β1–induced fibrotic response. There is increasing evidence that RSV also has the effect of cardiovascular protection and promoting the left ventricular function to recover [19, 20]. Furthermore, clinical research also showed that RSV improved diastolic function in the patients with coronary artery disease [21]. However, the effects and mechanisms of RSV on myocardial fibrosis in DCM are still unclear.

In this study, in order to investigate the effects and mechanisms of RSV on myocardial fibrosis, the rat model of DCM was used and the myocardial fibrosis with or without RSV intervention was assessed.

## Materials and methods

### Animals

Male specific pathogen free (SPF) Lewis rats (weight range 200–250 g, License No. SCXK (Beijing) 2012-0001) were purchased from Weitong Lihua Laboratory Animal Technology Co., Ltd. (Beijing, PR China) and maintained in the Laboratory Animal Center, Fujian Medical University, PR China. Animal care and experimental protocol were approved by the special committee on Animal Welfare of Fujian Medical University (Approval number: 201598).

### Induction of DCM

As previously described, each rat was immunized twice to induce DCM [22], with some modifications. To obtain a final concentration of 5 mg/ml, the porcine cardiac myosin (M-0531, Sigma Chemicals, St Louis, MO, USA) was mixed and fully emulsified with complete Freund’s adjuvant(1:1, v/v) supplemented with Mycobacterium tuberculosis H37Ra (Sigma Chemicals, St Louis, MO, USA). On day 0 and day 7, the emulsified solution (0.2 ml) was subcutaneously injected into the bilateral hind footpads of rats. In addition to the above operations, the adjuvant alone was simultanously subcutaneously injected into the same body part of rats in the control group.

### RSV intervention

DCM rats were divided into three groups (DCM, RSV-L and RSV-H groups) including 5 rats each without any artificial restrictions. The solutions were obtained by dissolving normal saline with RSV (Sigma, St Louis, MO, USA) at concentrations of 1 mg/ml and 5 mg/ml. Rats were exposed to RSV diluents 10 mg/kg in RSV-L group and 50 mg/kg in RSV-H group by oral gavage once daily, from the 28th day till the 90th day after the first immunization. The 90th day time point was selected according to the time course of collagen type I (Col I) and collagen type III (Col III) expressions in the pre-expreiment. During the same period, the same volume of normal saline was administrated in the DCM and control groups. All animals were killed on day 90 after echocardiography. Body weight and heart weight were measured. Every heart was divided into two parts along the coronal plane. One part of heart was fixed in 10% formaldehyde for 24 h in order to stain with hematoxylin–eosin (H&E), Masson and picrosirius red, the other part was immediately frozen in liquid nitrogen until used.

### Echocardiographic analysis

Transthoracic echocardiographic analysis was performed using a Vevo 770 ultrasound system (Visual Sonics Inc, Toronto, ON, Canada) with a 17.5 MHz imaging transducer on the 90th day after immunization. Anesthesia of rat was induced by intraperitoneal administration of Chloral hydrate (10%, 30 mg/kg, Ouenruisi chemical reagent co. LTD, Chengdu, PR China). M-mode echocardiography was performed at the level of the chordae tendineae, and the left ventricular end-diastolic diameter (LVEDD), the left ventricular end systolic diameter (LVESD), left ventricular posterior wall thickness (LVPWT), and left interventricular septal thickness (LIVST) were measured; left ventricular ejection fraction (LVEF) and left ventricular shortening fraction (LVSF) were also calculated.

### Histopathological examination

The rats were sacrificed, and each part of every heart was fixed in 3.8% perfusion of formaldehyde. After specimens were embedded in paraffin, they were sectioned into 5-μm slices. The sections were stained by the hematoxylin–eosin (H&E), Masson and picrosirius red, respectively. The morphology of cardiomyocyte and the deposition of fibrous tissues in the hearts were evaluated by the microscope. The percentage of collagen volume fraction (CVF) was performed with Image-pro Plus 6.0 software (Media Cybernetics, Bethesda, MD, USA) after staining by Masson. The percentage of collagen volume fraction, that is, the collagen area/total area ratio was calculated.

### Quantitative real-time polymerase chain reaction (qRT-PCR)

To extract the total RNA, the preserved heart was homogenized and the tissue was prepared by Trizol (Invitrogen, Carlsbad, CA, USA). The synthesis of the first strand of complementary DNA (cDNA) was based on the manufacturer’s instructions and a PrimeScript 1st strand cDNA synthesis kit (TaKaRa Bio, Inc., Otsu, Shiga, Japan) was used. The mRNA levels of collagen type I (Col I), collagen type III (Col III) and silence information regulator 1 (Sirt1) were measured by qRT-PCR with SYBR green (TaKaRa Bio, Inc., Otsu, Shiga, Japan) as the detected fluoroprobe. The reaction mixture (20 μL) consisted of cDNA from 100 ng of total RNA, 10 μM of each primer showed in Table 1, and 10 μL 2 × SYBR. β-actin played the role as an internal control in qRT-PCR. The relative expression (2^−ΔΔCT^) was calculated to evaluate the mRNA levels of Col I, Col III, and Sirt1.

**Table 1:** Primer sequences for quantitative real-time polymerase chain reaction (qRT-PCR)

	Accession number	Primer sequence (5′–3′)	Fragment sizes (bp)
Col I	NM_007743	Forward: GTGCAGTCGGTGCTCCAG	95
		Reverse: TTCTCCTTTGCCTCCAGGTATG	
Col III	NM_009930	Forward: CCTCTCTTATTTTGGCACAGCA	103
		Reverse: TGACATGGTTCTGGCTTCCA	
Sirt1	NM_019812	Forward: TTGGCACCGATCCTCGAA	217
		Reverse: ACAGAAACCCCAGCTCCA	
β-actin	NM_007393	Forward: CCCATCTACGAGGGCTATGC	150
		Reverse: TTTGATGTCACGCACGATTTC	

Col I, collagen type I; Col III, collagen type III; Sirt1, Silence information regulator1

### Coimmunoprecipitation

For coimmunoprecipitation, the heart tissue lysates were obtained by homogenization in cell lysis buffer for western and immunoprecipitation (Beyotime Biotechnology, Shanghai, PR China) supplemented with 1 mM phenylmethylsulphonyl fluoride (PMSF) and phosphatase inhibitor cocktails (Beyotime Biotechnology, Shanghai, PR China) on ice. Subsequently, the solution was centrifuged at 15,000 g for 10 min at 4 °C to clear the lysates. Protein concentration was determined according to the manufacturer’s instructions by BCA Protein Assay Kit (Beyotime Biotechnology, Shanghai, PR China). The lysates containing 500 μg proteins incubated with 20 μL Rabbit anti-Smad3 antibody (ab227223, Abcam, USA) for 2 h at 4 °C on a rocking platform. Then agarose A/G beads (Santa Cruz Biotechnology) and the immune complexes were completely mixed and incubated over night at 4 °C with rocking. The immunoprecipitates were collected by centrifugation and washed three times with phosphate buffer saline buffer. After being boiled in SDS sample buffer (0.2 M Tris–HCl, pH 6.8, 20% glycerol, 0.05% bromphenol blue, 7.72 mg/ml DTT) for 5 min, the immunoprecipitates were subjected to SDS gel electrophoresis and Western blotting. Anti-Smad3 antibody (Abcam), Anti-Sirt1 antibody, Anti-Acetylated-Lysine antibody and Anti-Phospho-Smad3 (Ser423/425) antibody which purchased from Cell Signaling Technology (Beverly, MA, USA) were used to detect Smad3, Sirt1, Acetylation Smad3 (Ac-Smad3) and Phospho-Smad3 (p-Smad3), respectively.

### Statistical analysis

All data were expressed as mean ± SD and analyzed with SPSS version 16.0 (SPSS Inc., Chicago, IL, USA). One-way analysis of variance (ANOVA) test and student’s t test among groups were selected as the methods for analyzing the differences of the data. A two-sided P value < 0.05 was considered as significant.

## Results

### Cardiac function was rescued by RSV

**Fig.1:**
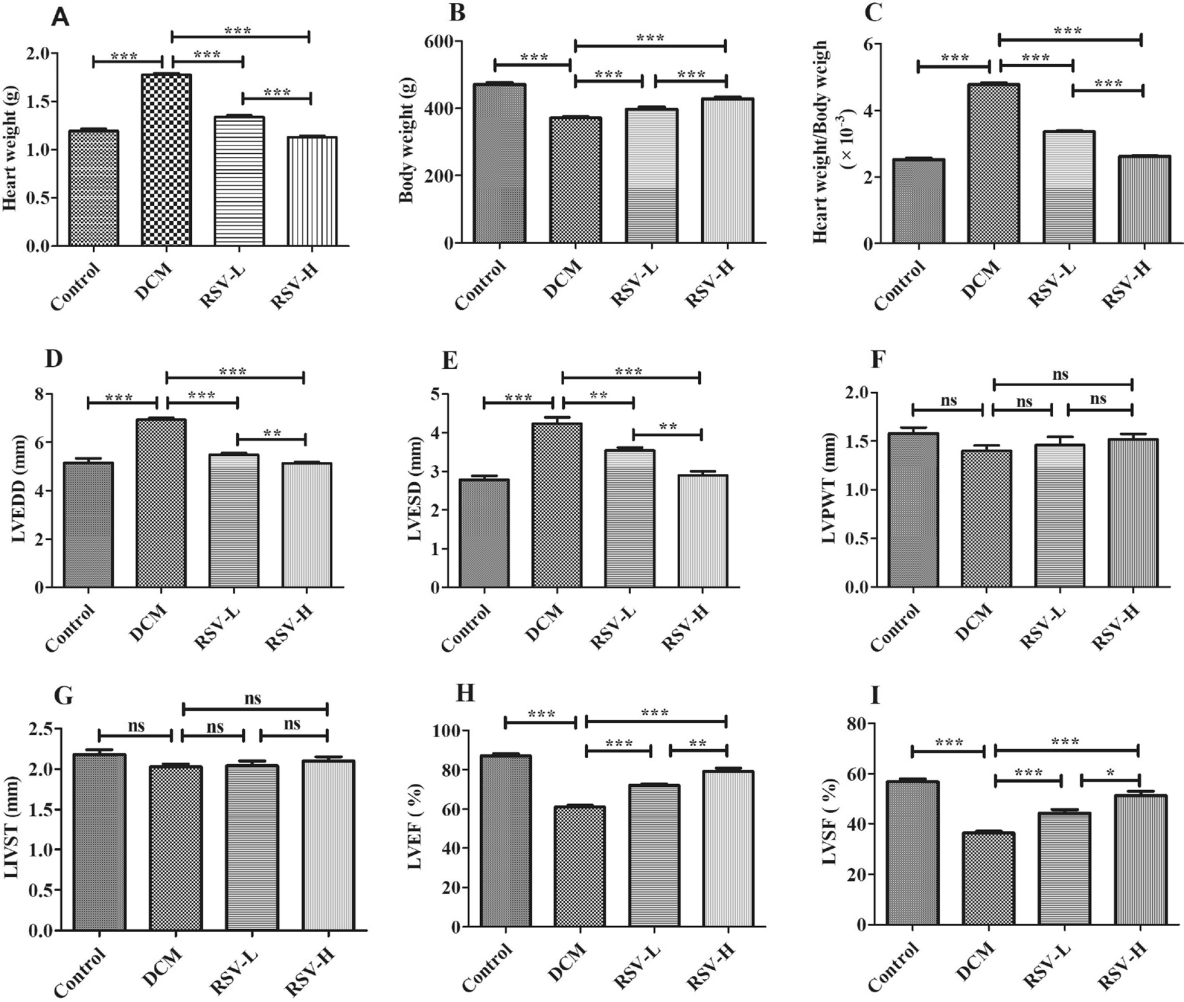
Cardiac function was rescued by RSV. All rats (5 rats in each group) were survived and the effects of RSV on cardiac function were evaluated using echocardiography on the ninetieth day after immunization. Our results showed that myocardial dilation and cardiac hypertrophy were significantly ameliorated and cardiac function was enhanced by RSV in rats with DCM. **a** Heart weight, **b** body weight, **c** heart weight/body weight, **d** LVEDD, Left ventricular end diastolic diameter, **e** LVESD, left ventricular end systolic diameter; **f** LVPWT, Left ventricular posterior wall thickness; **g** LIVST, Left interventricular septal thickness; **h** LVEF, Left ventricular ejection fraction; **i** LVSF, Left ventricular shortening fraction. RSV, Resveratrol; DCM, dilated cardiomyopathy disease; RSV-L, low dose of RSV (10 mg/kg/d); RSV-H, high dose of RSV (50 mg/kg/d). Data were presented as mean ± SD. ***, *P* < 0.0001; **, *P* < 0.001; *, *P* < 0.05; ns, *P* > 0.05

All rats (5 rats in each group) were survived on the ninetieth day after immunization. Our results showed that heart weight, heart weight/body weight ratio, LVEDD and LVESD were significantly higher in rats with DCM than controls (all *P* < 0.0001), while body weight, LVEF and LVSF were obvious lower in rats with DCM when compared to controls (all *P* < 0.0001). After intervention with RSV, heart weight, heart weight/body weight ratio, LVEDD and LVESD were significantly decreased while body weight, LVEF and LVSF were significantly increased (RSV-L or RSV-H vs. DCM, all *P* < 0.001). Moreover, the intervention effect of RSV was dose-dependent (RSV-L vs. RSV-H, *P* < 0.05) (Fig. 1). Therefore, our data reflected that RSV has the effect of ameliorating myocardial dilatation, enhancing myocardial contractility and improving cardiac function in rats with DCM, though there were no differences in LVPWT and LIVST among groups.

### Deposition of fibrous tissues in the hearts were decreased and myocardial intercellular gap was narrowed by RSV in rats with DCM

**Fig. 2:**
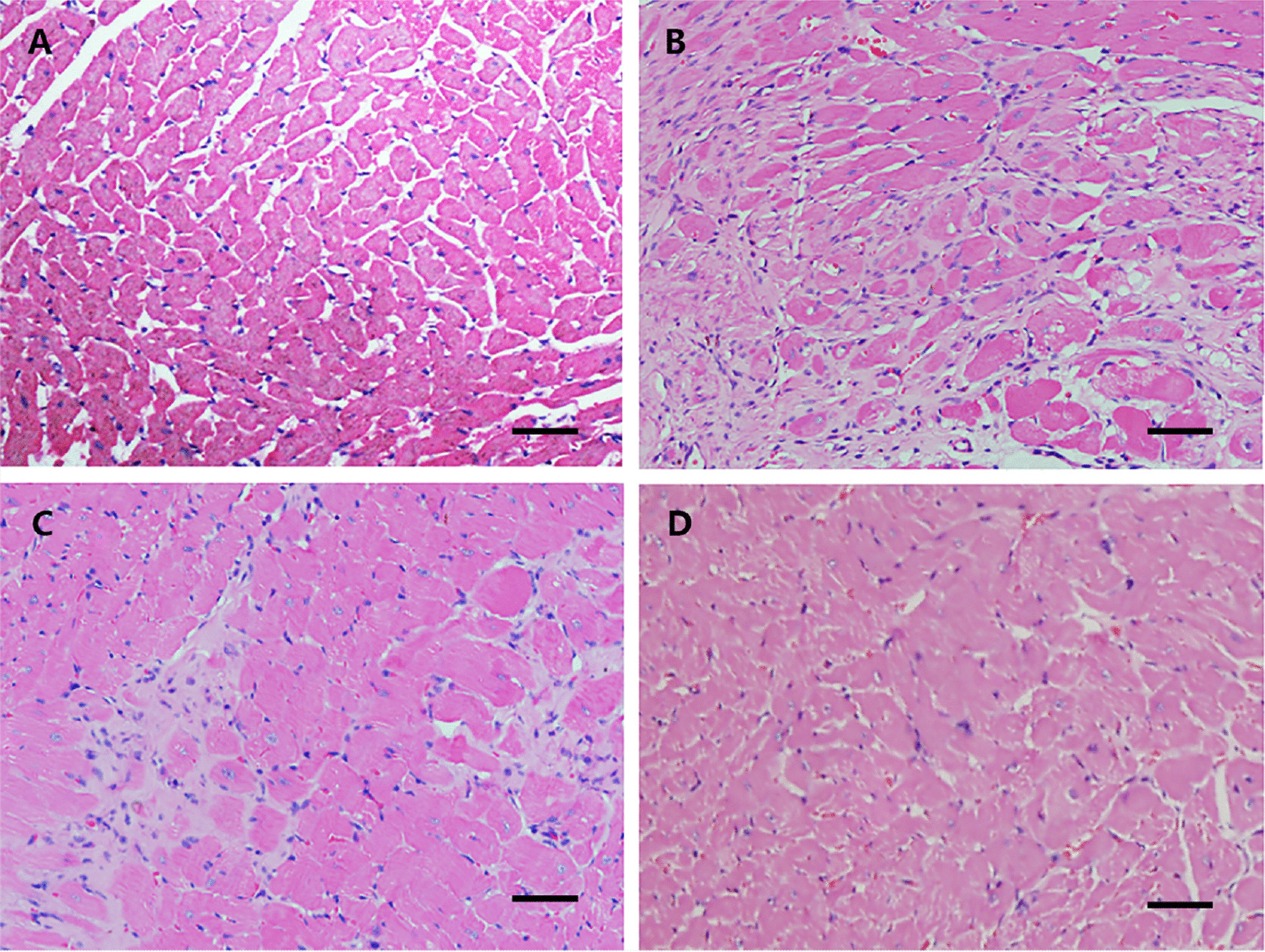
Deposition of fibrous tissues in the hearts were decreased by RSV in rats with DCM. Rats were sacrificed on the ninetieth day after the first immunization. The myocardial tissues were stained with H&E. Representative images of **a** control group,**b** DCM group, **c** RSV-L group, **d** RSV-H group were shown. RSV, Resveratrol; DCM, dilated cardiomyopathy disease; RSV-L, low dose of RSV (10 mg/kg/d); RSV-H, high dose of RSV (50 mg/kg/d); H&E: Hematoxylin–eosin. Scale bar 50 μm

To observe the morphology of cardiomyocyte and the deposition of fibrous tissues in the hearts, myocardial tissues were stained with H&E (Fig. 2). The hearts in the DCM group showed the features of disorderly arrangement and fibrosis. After intervention with RSV, the deposition of fibrous tissues was decreased in the RSV-L group and RSV-H group when compared to DCM group. Moreover, the arrangement of cardiomyocytes was close to normal and the gaps among cells were furtherly reduced in the RSV-H group compared to RSV-L group. Overall, RSV attenuated the fibrosis of the myocardium of rats with DCM.

### Collagen deposition in myocardium were significantly reduced by RSV in rats with DCM

**Fig. 3:**
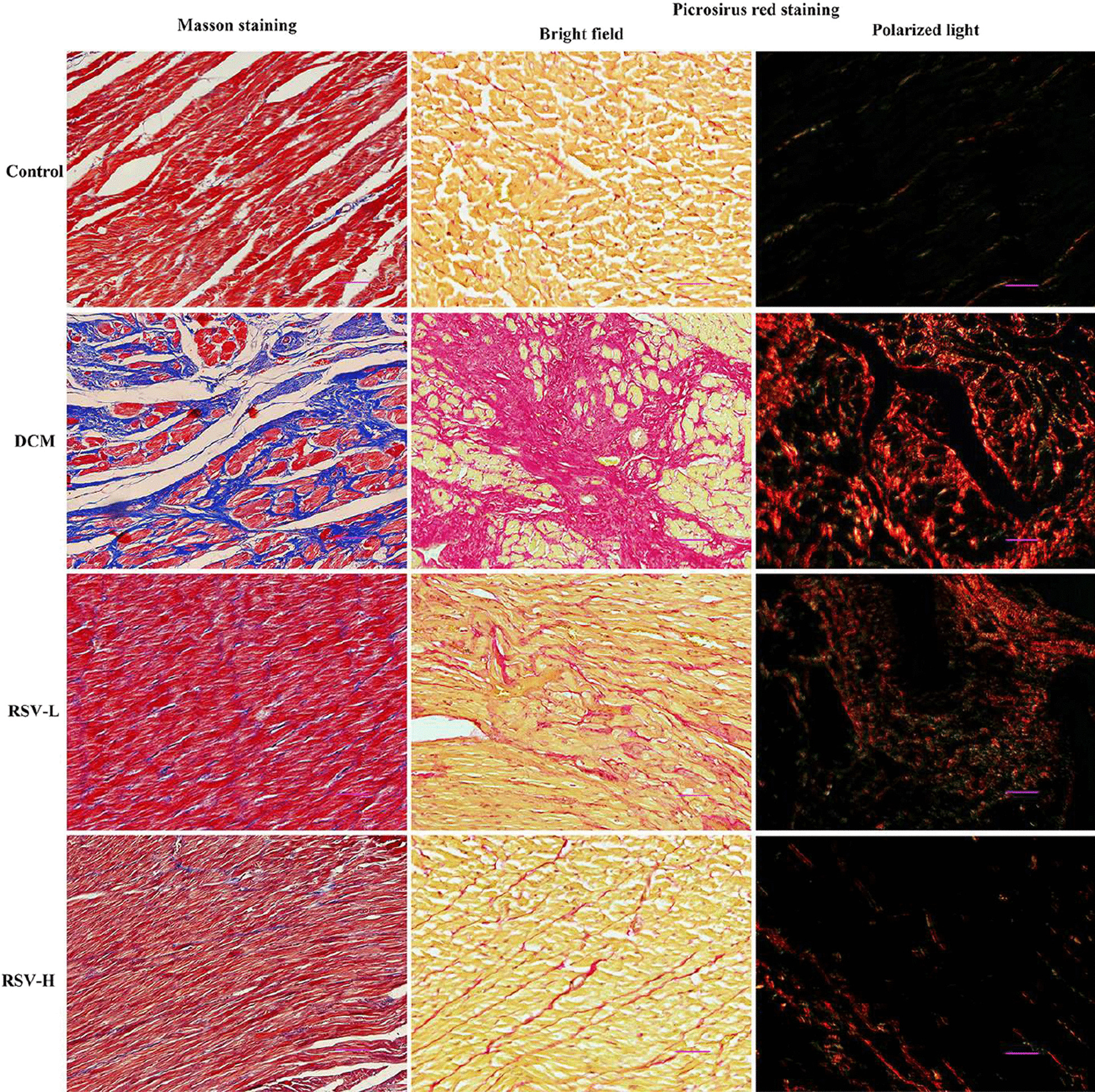
Collagen deposition in myocardium were significantly reduced by RSV in rats with DCM. The myocardial tissues were stained with Masson and picrosirius red. One representative image was shown for each group. Slides stained with picrosirius red were examined by bright field and polarized light. After staining with Masson, there was a large amount of collagen fibers (blue) disorderly deposited between myocardial cells in the DCM group, while there was only a very small amount of collagen fibers observed in the control group. Under bright field, a large amount of collagen fibers (red) deposition were also observed in the DCM group and they were decreased by RSV in the RSV-L and RSV-H groups. Under polarized light, collagen fibers type I (yellow–red staining) was markedly increased in the DCM group compared to the control group. After the intervention with RSV, collagen tissues were decreased in the RSV-L and RSV-H groups. RSV, Resveratrol; DCM, dilated cardiomyopathy disease; RSV-L, low dose of RSV (10 mg/kg/d); RSV-H, high dose of RSV (50 mg/kg/d). Scale bar 50 μm

To further evaluate the effect of RSV on the deposition of collagen fibers, cardiac tissues were stained with Masson and picrosirius red (Fig. 3). Our results showed that collagen fibers were mainly distributed around the blood vessels and only a small amount of collagen fibers were found in the control group. However, in DCM group, there was a large amount of collagen fibers disorderly deposited between myocardial cells. Collagen fibers were significantly decreased after RSV intervention in the RSV-L and RSV-H groups. Moreover, compared to the RSV-L group, the amount of collagen was further decreased and closed to normal in the RSV-H group.

### The mRNA levels of Col I and Col III were decreased, while the mRNA levels of Sirt1 were increased by RSV in rats with DCM

**Fig. 4:**
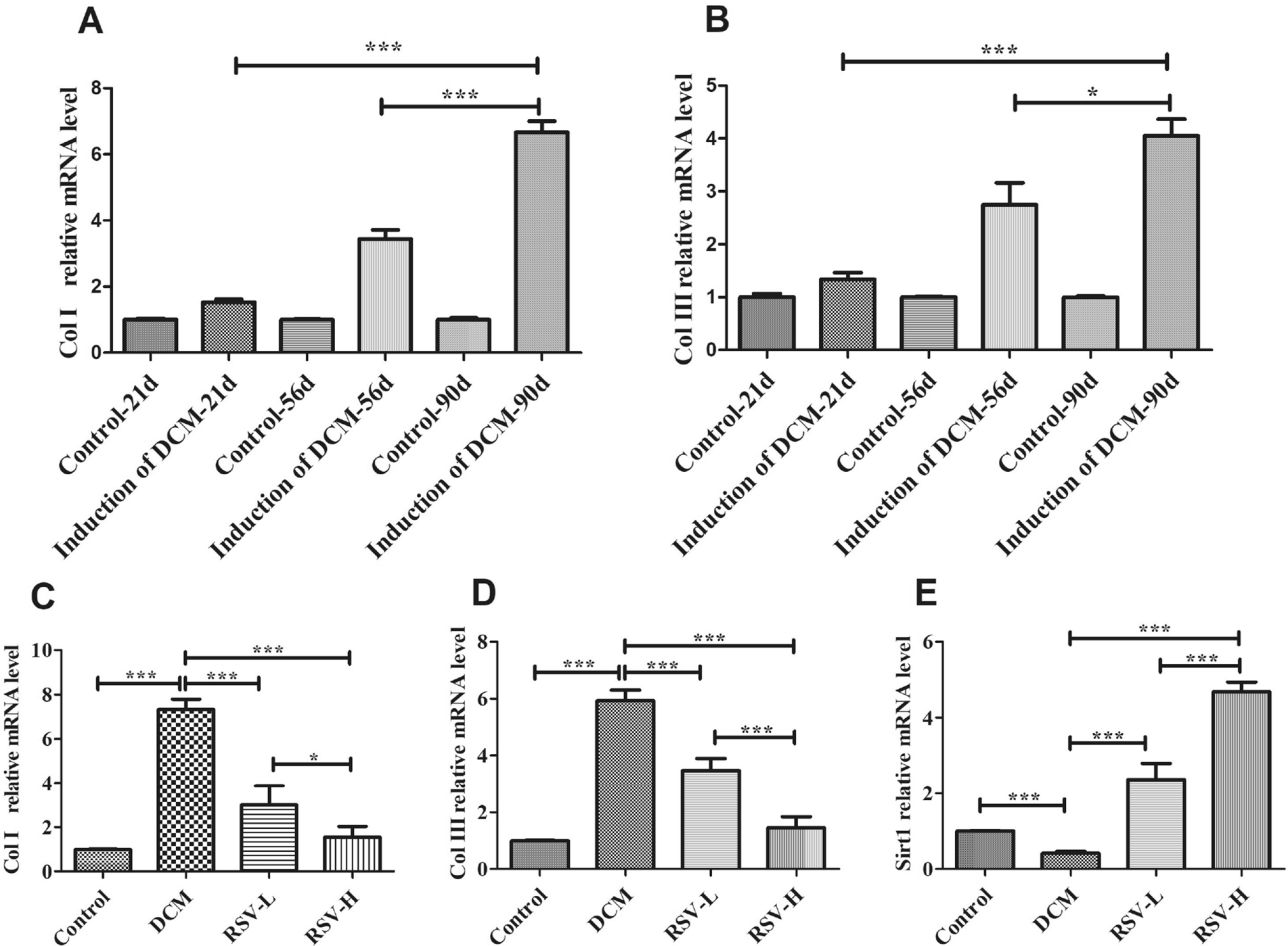
The mRNA levels of Col I and Col III were decreased, while the mRNA levels of Sirt 1 were increased by RSV in rats with DCM. The mRNA levels of Col I, Col III and Sirt1 were measured by qRT-PCR. β-actin was amplified as an internal control. The fold change (2^−ΔΔCT^) was calculated to evaluate the mRNA levels. Values were presented as mean ± SD. **a**, **b** The mRNA levels of Col I, Col III in the heart on the 21st, 56th, 90th day after the first immunization. **c**–**e** The mRNA levels of Col I, Col III and Sirt1 in the heart on the 90th day after the first immunization. Col I, collagen type I; Col III, collagen type III; Sirt1, Silence information regulator 1; DCM, dilated cardiomyopathy; RSV-L, low dose of RSV (10 mg/kg/d); RSV-H, high dose of RSV (50 mg/kg/d); ***, *P* < 0.0001; *, *P* < 0.05

In order to further verify the effect of RSV on the expressions of Col I and Col III, the mRNA levels of Col I, Col III and Sirt1 were examined and shown in Fig. 4. Col I and Col III in the hearts showed higher expression in groups compared with control group (*P* < 0.0001). After intervention with RSV, the expression of them was significantly decreased compared to the DCM group (*P* < 0.0001). Moreover, the mRNA levels of Col I and Col III in the RSV-H group were lower than RSV-L group (*P* < 0.05 and *P* < 0.0001, respectively). On the contrary, lower the Sirt1 mRNA level was detected in the DCM group rather than the control group. But it was positively increased after intervention by RSV in the RSV-L and RSV-H groups compared to DCM group (*P* < 0.0001). Moreover, the Sirt1 mRNA levels in the RSV-H group were higher than RSV-L group (*P* < 0.0001). Our results reflected that RSV can decrease the expression of Col I and Col III and increase the expression of Sirt1 in the rats with DCM.

### AC-Smad3, not p-Smad3 was mainly down regulated by Sirt1

**Fig. 5:**
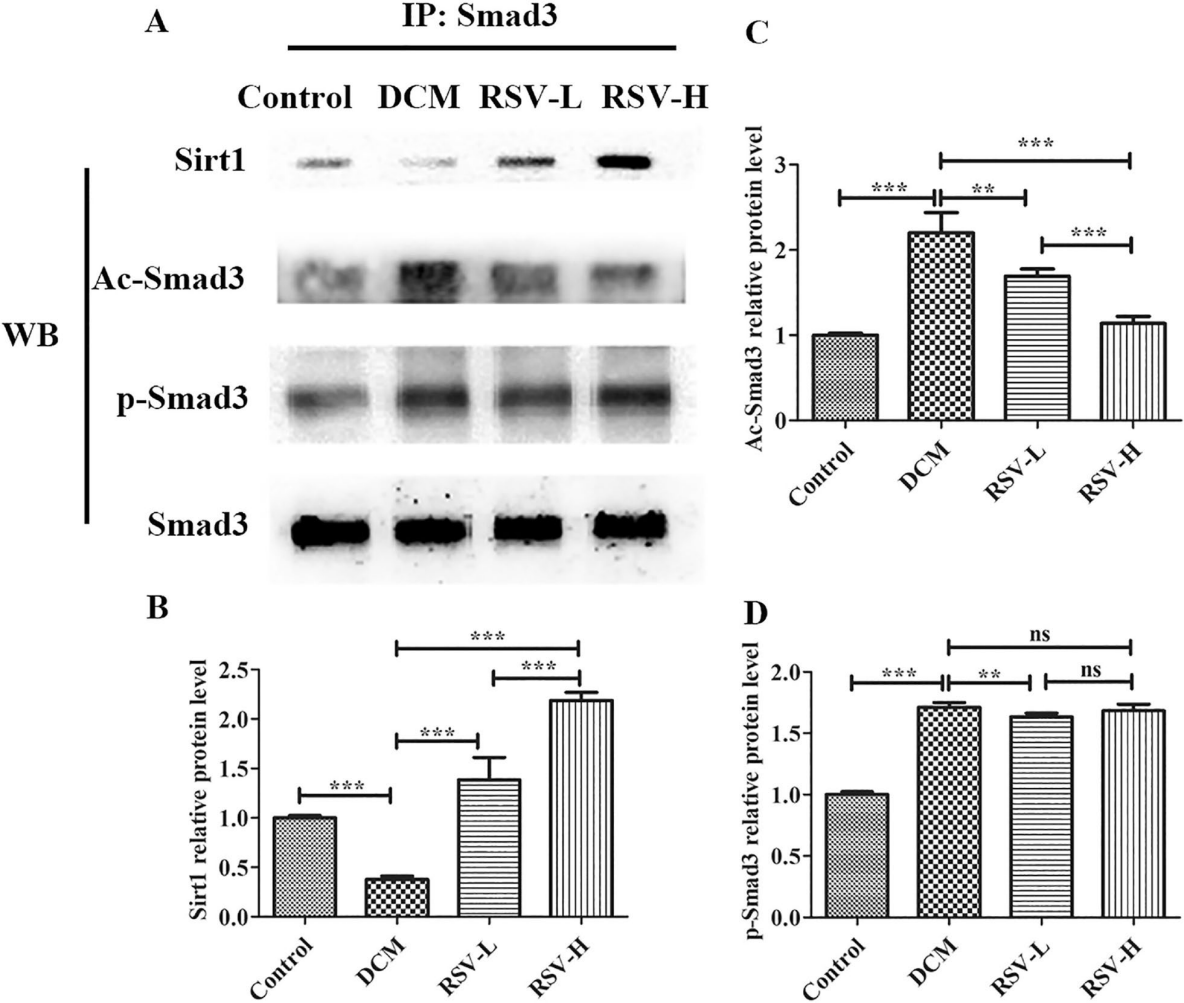
AC-Smad3, not p-Smad3 was mainly down regulated by Sirt1. The interaction between Sirt1 and Smad3 was detected by immunoprecipitation. **a** One representative image of immunoprecipitation. **b**–**d** Statistical analysis for the relative protein levels of Sirt1, AC-Smad3 and p-Smad3. AC-Smad3, Acetylation Smad3; p-Smad3, phospho-Smad3; Sirt1, Silence information regulator1; DCM, dilated cardiomyopathy; RSV-L, low dose of RSV (10 mg/kg/d); RSV-H, high dose of RSV (50 mg/kg/d); ***, *P* < 0.0001; **, *P* < 0.001; ns, *P* > 0.05

In order to confirm whether the activated Sirt1 directly combines with Smad3, the immunoprecipitation assay was used. Sirtl was detected in the protein complex connected to the anti-smad3 antibody by western blot, which confirmed the direct combination between Smad3 and Sirt1 (Fig. 5). Our results also showed that Sirtl in the DCM group showed significant lower expression than that in the control group (*P* < 0.0001), while the expression of Sirt1 was significantly increased after RSV intervention (RSV-L or RSV-H vs DCM, *P* < 0.0001), with dose-dependent effect (RSV-L vs RSV-H, *P* < 0.0001). Contrary to Sirtl, a significant higher expression of Ac-smad3 in the DCM group compared to the control group was observed (*P* < 0.0001). After RSV intervention, Ac-smad3 was significantly decreased (RSV-L vs DCM, *P* < 0.001; or RSV-H vs DCM, *P* < 0.0001), also with dose-dependent effect (RSV-L vs RSV-H, *P* < 0.0001). Although phospho-Smad3 (p-Smad3) in the DCM group was also significantly higher compared to the control and showed a slight decrease after low dose of RSV intervention, there was no dose effect (RSV-L vs DCM, *P* > 0.05) and significant difference between RSV-H group and DCM group. Therefore, our data reflected that Sirt1 can be increased by RSV, and the combination of Sirt1 with Smad3 will mediate the deacetylation of Ac-smad3 and promote the repair of myocardial fibrosis in rats with DCM.

## Discussion

This study illustrated the effects of resveratrol (RSV) on rats with dilated cardiomyopathy (DCM). The study showed that RSV ameliorated the cardiac function of rats with DCM and attenuated the expressions of collagen type I (Col I) and collagen type III (Col III). The amelioration of cardiac function and myocardial fibrosis was associated with an activation of silence information regulator 1 (Sirt1) which can directly combine with Smad3 and promote its deacetylation.

RSV effectively ameliorates cardiac hypertrophy and increase cardiac contractility in rats with DCM. In agreement with YoshidaY et al. [23], this study showed that the heart weight, heart weight/body weight ratio, LVEDD and LVESD were significantly decreased while body weight, LVEF and LVSF were significantly increased after intervention with RSV. Our study also reveals that RSV has the effect of attenuating myocardial dilatation and improving cardiac function.

RSV is a natural activator for Sirt1 [18]. Consistent with this theory, the mRNA level of Sirt1 in the myocardial tissues was increased by RSV. And our results also showed that the degree of increase was related to the dosage of RSV. But the Sirt1 mRNA was decreased in the DCM group which was only immunized by myosin. It was different from the study conducted by YoshidaY et al.[23] which reported that the Sirt1 mRNA was increased in the myocardium of myosin—immunized rats, but not in the myocardium of RSV or both RSV and myosin intervention. This difference may be related to the different inflammatory stages detected in the two studies.

Myocardial fibrosis is the main characteristic of DCM. This study found that the deposition and mRNA levels of fibrous tissues including Col I and Col III in the hearts were decreased by RSV in rats with DCM. Moreover, the effect on the reduction of Col I and Col III mRNAs was related to the dosage of RSV. Therefore, RSV also can decrease myocardial fibrosis in rats with DCM. Study had found that the over expression of Sirt1 can reduce myocardial hypertrophy and interstitial fibrosis [24]. Thus, the reduction of Col I and Col III may be related to RSV administration. The formation of myocardial fibrosis is the result of increasing collagen synthesis and/or decreasing collagen degradation. The expression and activity of matrix metalloproteinase may also affect the formation of myocardial fibrosis. RSV was also able to change the expression and activity of matrix metalloproteinase 2 [7].

Sirt1 is a member of histone deacetylase which can deacetylate a variety of proteins including Smad3 [25]. Our results also reflected that the activated Sirt1 directly combines with Smad3 and down regulated the acetylation of Smad3 (Ac-Smad3) but not phospho-Smad3 (p-Smad3) by RSV in the rats with DCM. Li J, et al.[9] found that RSV can inhibit renal fibrosis by the activation of Sirt1, which mediated the deacetylation of Smad3 and suppressed the TGF-β1–induced fibrotic response. The activated SIRT1 also had the effect of preventing cardiac fibrosis by suppressing the activation of Smad3 acetylation [8]. Thus Sirt1/Smad3 deacetylation pathway may also be involved in the development of myocardial fibrosis. With the decrease of Ac-Smad3, the Col I and Col III mRNAs were also decreased in our study. Therefore, our data reflect that RSV also has a decreasing effect on the collagen fibers by activating Sirt1 which mediate the deacetylation of Ac-smad3 in the rats with DCM.

Additionally, RSV was administered to rats with DCM via oral gavage which is closer to oral administration than intraperitoneal injection. Our results showed that myocardial fibrosis was effectively attenuated and cardiac function was significantly improved by oral gavage of RSV, which provides a laboratory basis for the clinical application of RSV.

This study had some limitations. First, the effect of resveratrol on preventive treatment has not been explored. Second, the dynamic evaluation of cardiac function by echocardiographic analysis was also lack. It was impossible to know the changes of cardiac function real time during the administration of resveratrol. Third, Sirt1 gene knockout or over expression rats were not involved in the study. Fourth, the sample size selected in this study was not full calculation and just according to the sample size used in the related references. Therefore, the detailed and precise interaction between resveratrol and Sirt1 remains to be further studied. In addition, our results were obtained from an animal model and further clinical study is still essential.

## Conclusion

Rresveratrol effectively ameliorated myocardial fibrosis and improved cardiac function by regulating Sirt1/Smad3 deacetylation pathway in the rat model with dilated cardiomyopathy. Resveratrol may be a therapeutic modality for ameliorating myocardial fibrosis and improving cardiac function for patients with dilated cardiomyopathy.

## Data Availability

The datasets generated and analysed during the current study are not publicly available due the principle of funding confidentiality but are available from the corresponding author upon reasonable request.

## Abbreviations

Ac-Smad3: Acetylation of Smad3
cDNA: Complementary DNA
Col I: Collagen type I
Col III: Collagen type III
CVF: Collagen volume fraction
DCM: Dilated cardiomyopathy
H&E: Hematoxylin–eosin
LVEDD: Left ventricular end diastolic diameter
LVESD: Left ventricular end systolic diameter
PMSF: Phenylmethylsulphonyl fluoride
p-Smad3: Phospho-Smad3
qRT-PCR: Quantitative real-time polymerase chain reaction
RSV: Resveratrol
Sirt1: Silence information regulator 1
SPF: Specific pathogen free
TGF-β1: Transforming growth factor-beta 1
